# Comparing Web-Based Provider-Initiated and Patient-Initiated Survivorship Care Planning for Cancer Patients: A Randomized Controlled Trial

**DOI:** 10.2196/cancer.5947

**Published:** 2016-08-30

**Authors:** Katherine Clegg Smith, Elliott Tolbert, Susan M Hannum, Archana Radhakrishnan, Kelsey Zorn, Amanda Blackford, Stephen Greco, Karen Smith, Claire F Snyder

**Affiliations:** ^1^ Johns Hopkins Bloomberg School of Public Health Baltimore, MD United States; ^2^ Sidney Kimmel Comprehensive Cancer Center Baltimore, MD United States; ^3^ Johns Hopkins School of Medicine Baltimore, MD United States; ^4^ Sidney Kimmel Comprehensive Cancer Center Division of Biostatistics and Bioinformatics Baltimore, MD United States; ^5^ Johns Hopkins School of Medicine Department of Radiation Oncology Baltimore, MD United States; ^6^ Suburban Hospital Bethesda, MD United States; ^7^ Sibley Memorial Hospital Washington, DC United States; ^8^ Johns Hopkins School of Medicine Department of General Internal Medicine Baltimore, MD United States; ^9^ Johns Hopkins Bloomberg School of Public Health Department of Health Policy and Management Baltimore, MD United States

**Keywords:** survivorship care plan, mixed methods study, randomized controlled trial

## Abstract

**Background:**

Survivorship care plans (SCPs) are intended to facilitate communication and coordination between patients, oncologists, and primary care providers. Most SCP initiatives have focused on oncology providers initiating the SCP process, but time and resource barriers have limited uptake.

**Objective:**

This trial compares the feasibility and value of 2 Web-based SCP tools: provider-initiated versus patient-initiated.

**Methods:**

This mixed-methods study recruited clinicians from 2 academically-affiliated community oncology practices. Eligible patients were treated by a participating oncologist, had nonmetastatic cancer, completed acute treatment ≤ 2 months before enrollment, and had no evidence of disease. Patients were randomized 1:1 to either provider-initiated or patient-initiated SCPs—both are Web-based tools. We conducted qualitative interviews with providers at baseline and follow-up and with patients 2 months after enrollment. In addition, patients were administered the Preparing for Life as a (New) Survivor (PLANS) and Cancer Survivors’ Unmet Needs (CaSUN) surveys at baseline and 2 months.

**Results:**

A total of 40 providers were approached for the study, of whom 13 (33%) enrolled. Providers or clinic staff required researcher assistance to identify eligible patients; 41 patients were randomized, of whom 25 completed follow-up (61%; 13 provider-initiated, 12 patient-initiated). Of the 25, 11 (44%) had initiated the SCP; 5 (20%) provided the SCP to their primary care provider. On the Preparing for Life as a (New) Survivor and Cancer Survivors’ Unmet Needs, patients in both arms tended to report high knowledge and confidence and few unmet needs. In qualitative interviews, providers and patients discussed SCPs’ value.

**Conclusions:**

Regardless of patient- versus provider-initiated templates and the Web-based design of these tools, barriers to survivorship care planning persist. Further efforts should emphasize workflow *functions* for identifying and completing SCPs—regardless of the SCP *form* used.

**Trial Registration:**

ClinicalTrials.gov NCT02405819; https://clinicaltrials.gov/ct2/show/NCT02405819 (Archived by WebCite at http://www.webcitation.org/6jWqcWOvK)

## Introduction

The completion of active cancer treatment is a critical juncture when patients need support and communication to ensure optimal health and quality of life outcomes. The 2005 Institute of Medicine (IOM) report “ *From Cancer Patient to Cancer Survivor: Lost in Transition”* [1] highlighted the difficulty that many cancer patients face when transitioning from acute treatment. The IOM report recommended that patients completing treatment receive a summary of the treatments received and a plan for follow-up care. These materials have become known as a “survivorship care plan” (SCP). SCPs have become a target initiative for patient-centered improvements to oncology, but the literature on their implementation and impact remains sparse and inconclusive [2-8].

Based on the IOM recommendation, various organizations have developed SCP templates. Most of these templates have been designed with the intention of oncology providers initiating the survivorship care planning process. Uptake of survivorship care planning has, however, been slow and limited [9]. There are now several initiatives underway that are reconsidering survivorship care planning approaches, updating available templates, or both [10-12]. Web-based, patient-initiated SCPs are one alternative whereby the patient is empowered to at least begin completion of a treatment summary and care plan at home. The idea behind the patient-initiated approach is that this may serve to reduce barriers related to available time and resources in the oncology clinic, while further engaging patients in self-care. Journey Forward is a collaboration of the National Coalition for Cancer Survivorship, the UCLA Cancer Survivorship Center, the Oncology Nursing Society, Anthem Inc., and Genentech [12]. The Journey Forward collaboration has developed both the “Survivorship Care Plan Builder” (provider-initiated; see Multimedia Appendix 1) and “My Care Plan” (patient-initiated; see Multimedia Appendix 2) Web-based templates and has made these tools freely available on the Web. It is also possible to print the forms and fill them out by hand.

In this study (ClinicalTrials.gov NCT02405819), we sought to compare the feasibility and value of the 2 Journey Forward models of SCP provision. We designed the study to provide initial evidence of the feasibility and possible value of 2 models of SCP provision. We present data from the perspectives of both patients and providers regarding implementation processes and feasibility, facilitators and barriers, and perceived value of the survivorship care planning process.

## Methods

### Study Design

This mixed-methods study comparing 2 modalities of SCPs (“Care Plan Builder” and “My Care Plan”) used a randomized design and was conducted in 2 community-based, academically affiliated hospitals [13]. We recruited oncologists who manage breast, prostate, and colorectal cancer patients. Patient eligibility was not, however, limited to breast, prostate, and colorectal cancer; patient participants were recruited through the participating clinicians and were adults (21 years and older) diagnosed with any nonmetastatic cancer. Patients were enrolled in the study for a period of 4 months and were followed for 2 months. This study was reviewed and approved by Johns Hopkins School of Medicine Institutional Review Board.

### Clinician Participants

Participation of clinicians in the study was determined to be an indicator of feasibility, and we tracked the number of clinicians approached, the number eligible, and the number who consented to participate. Clinicians were approached to participate in the study through both in-person presentations of the work and through emailed requests. Consent to participate was acquired in-person. Once clinicians agreed to participate and provided written informed consent, we conducted a baseline qualitative interview in which we asked about experiences of survivorship care planning, expectations for the study, and the perceived value of SCPs. Once data collection with patients was complete, we conducted a follow-up interview with participating clinicians to ask about experiences with the interventions. In this interview, we revisited the issue of the perceived value of SCPs and obtained clinician feedback on the implementation (including barriers and facilitators) and feasibility of the 2 survivorship care planning approaches implemented in this trial.

### Patient Participants

To determine the feasibility of oncologists or oncology staff identifying patients for an SCP, the original study protocol called for participating oncologists to refer adult patients completing active treatment for nonmetastatic cancer to the study team. Specific patient eligibility criteria included having nonmetastatic disease, completed acute treatment within the past 2 months, and no evidence of disease. Although patients had to have completed acute treatment, patients on chronic treatment (>1 year) were eligible. Patient participants were identified in the clinic by clinic staff, and a member of the research team oversaw consent procedures. Eligible patients who agreed to participate provided written informed consent and were randomized 1:1 using a random number generator with the condition concealed until randomization; patients and their clinicians were then informed of the randomized condition. Patients were paid $35 for their participation in the study.

### Plan Initiation

For participants randomized to the patient-initiated My Care Plan group, the research team directed patients to the Web address for the appropriate tool and provided an instructional hand-out for reference. For participants randomized to the provider-initiated Survivorship Care Plan Builder, the provider was made aware of their randomization and was responsible for completing the SCP. The clinicians were all given information on the SCP Builder website or tool and were also familiarized with the patient-initiated My Care Plan tool.

### Data Collection and Outcome Measures

Data collection occurred at 2 time points: baseline and 2-month follow-up. The primary outcome was receipt of an SCP by the 2-month follow-up. Specifically, at the 2-month follow-up contact, we determined whether the patient had a partially or fully completed SCP versus no plan at all.

Secondary outcomes included supportive care needs assessed by the Cancer Survivors’ Unmet Needs (CaSUN) survey [14], and knowledge and confidence about survivorship assessed by the Preparing for Life as a (New) Survivor (PLANS) survey [15]. The CaSUN is a validated measure that includes 35 unmet need items with response options of no need or not applicable, met need, and weak, moderate, or strong unmet need. We assigned values of 1=no need or not applicable to 5=strong unmet need and used these to calculate means for the individual items. There are also 6 positive change items with response options of has always been like this, has been a positive outcome, no: want help to achieve this, and no: not important to me; these data are presented descriptively. The PLANS survey includes 11 knowledge items rated on a 4-point Likert scale from 1=strongly disagree to 4=strongly agree, as well as 5 confidence items rated on a 10-point scale from 1=not at all confident to 10=extremely confident. We calculated means for the individual PLANS items. These questionnaires, along with patient demographics, were collected by interviewer-assisted, patient report on paper forms, at baseline. The CaSUN and PLANS were also collected at the 2-month follow-up.

Finally, we conducted a brief, targeted qualitative interview with patients at follow-up regarding perceived impact of cancer, informational and support needs, as well as experiences and attitudes about the SCP tool to which they were randomized. This interview collected information on processes undertaken to complete the SCP (or challenges that prevented successful completion of an SCP), parts of the process the patients found helpful or that presented obstacles, and recommendations for improving the process.

Quantitative data from the CaSUN and PLANS were analyzed with summary statistics and descriptively by comparing the distribution of scores at baseline between intervention arms and the distribution of scores at follow-up between intervention arms using nonparametric Wilcoxon rank-sum tests. Changes from baseline to follow-up were described within interventions arms with Wilcoxon signed-rank tests. To describe the differential change from baseline to follow-up between intervention arms (ie, interaction), we compared the changes between intervention arms with Wilcoxon rank-sum tests. No formal sample size calculations were conducted for the secondary quantitative outcome measures; however, the results here can inform power calculations for future evaluations. Analysis of interview data from clinicians and patients was thematic and summative, with a focus on identification of perceived and experienced value of SCPs, as well as facilitators and barriers to implementation of both modalities. Interview data were read and reviewed by various members of the research team, with a view to establishing consensus about major emergent themes. All quantitative analyses were completed using statistical software R, version 3.3.0 [16].

## Results

### Survivorship Care Planning Feasibility: Provider and Patient Participation

Of the 40 eligible oncologists at the 2 hospitals, 13 (33%) agreed to participate in the study. Nearly half of the clinicians were female (46%); the sample included 5 radiation oncologists (38%), 5 medical oncologists (38%), and 3 surgeons (23%). The clinicians who did not choose to participate included 17 surgical oncologists and 10 medical oncologists; all eligible radiation oncologists chose to participate in the study. At the initiation of the study, none of the participating oncologists provided SCPs to patients as part of standard care. We conducted follow-up interviews with 11 of the 13 enrolled clinicians; 2 clinicians did not respond to numerous attempts to schedule an interview at follow-up.

Although the planned approach for patient recruitment was for oncologists and clinic staff to identify patients completing treatment, it became clear after 1 month of passive research observation that processes relying on the clinical teams were ineffective. For the remaining 3 months of recruitment, research staff worked with clinic staff to identify patients eligible for SCPs. A member of research staff was present on clinic days and reviewed schedules to identify potentially eligible patients who were due to have appointments. Research staff prompted clinic staff to discuss joining the study with potential participants.

**Figure 1:**
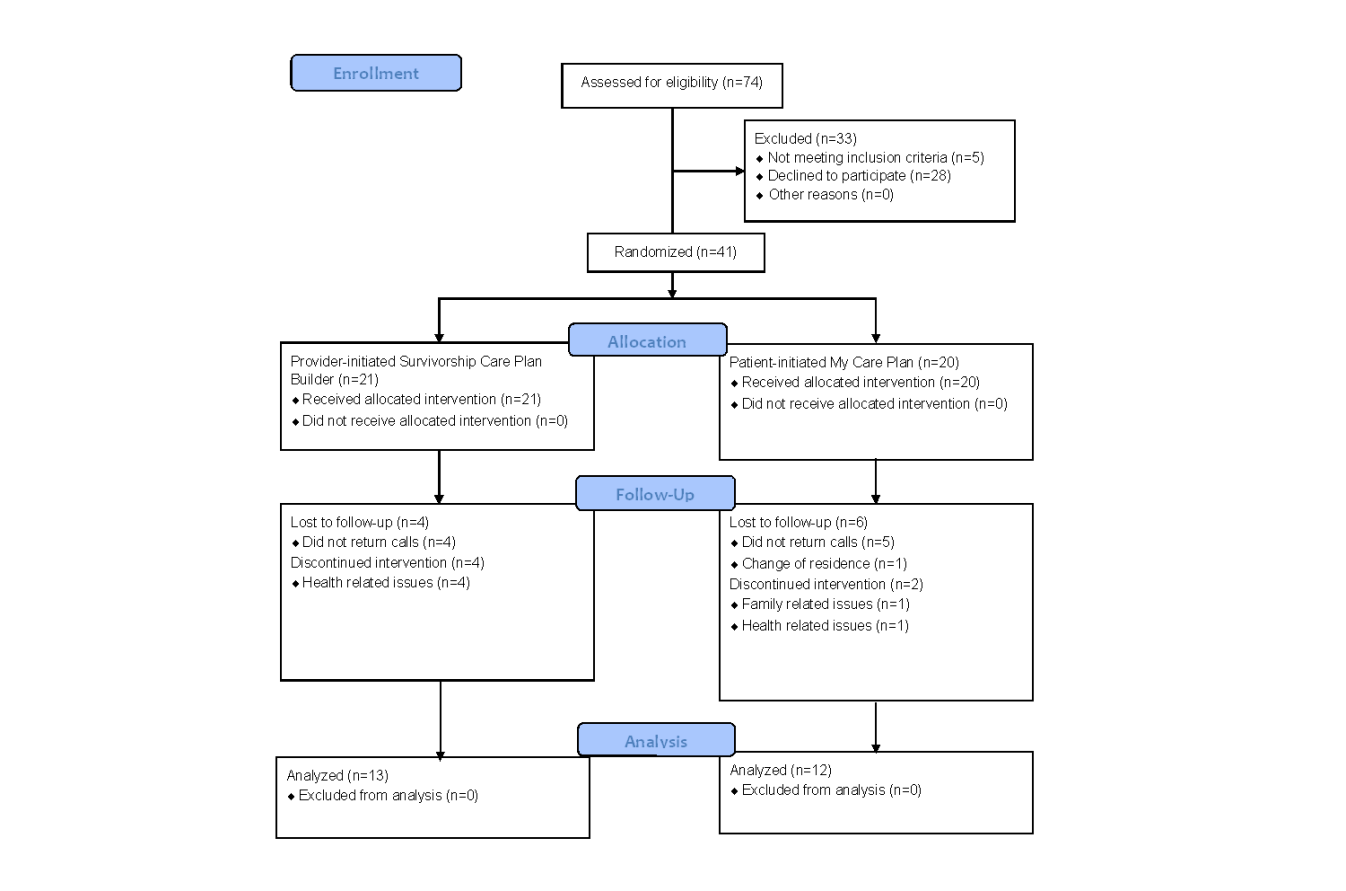
Enrollment into survivorship care planning trial.

In total, 74 patients were approached and 41 (55%) enrolled and were randomized—21 to the provider-initiated Survivorship Care Plan Builder and 20 to the patient-initiated My Care Plan (Figure 1). The 41 enrolled patients were recruited from 5 (38%) of the 13 participating clinicians; 3 of the referring clinicians were radiation oncologists, 1 a surgical oncologist, and 1 a medical oncologist. Participating patients were, on average, aged 66 years (range: 44-90 years), 68% female, 81% white, 59% married, and 51% reported excellent or very good health (Table 1). Breast cancer was the most common diagnosis (61%), followed by prostate cancer (20%), and lung cancer (10%). Patients were most commonly retired (46%) or working full-time (37%). Almost all participants had high-speed Web access (95%) and were regular computer users (85%).

**Table 1:** Patient characteristics overall and by study arm.^a^

Characteristic		All patients (N=41)	Provider-initiated (n=21)	Patient-initiated (n=20)
Age				
	Mean (Standard deviation)	66 (11.7)	66 (12.5)	65 (11.1)
	Median (Range)	63 (44-90)	64 (44-90)	62 (44-88)
Gender, n (%)				
	Male	13 (31.7)	6 (28.6)	7 (35.0)
Race, n (%)				
	White	33 (80.5)	16 (76.2)	17 (85.0)
	Black or African American	5 (12.2)	4 (19.0)	1 (5.0)
	Other	3 (7.3)	1 (4.7)	2 (10.0)
	Hispanic	5 (12.2)	2 (9.5)	3 (15.0)
Education, n (%)				
	High school graduate or lower	7 (17.1)	4 (19.0)	3 (15.0)
	Attended some college	3 (7.3)	2 (9.5)	1 (5.0)
	College graduate	8 (19.5)	3 (14.3)	5 (25.0)
	Any postsecondary work	23 (56.1)	12 (57.1)	11 (55.0)
Cancer type, n (%)				
	Breast	25 (60.9)	14 (66.7)	11 (55.0)
	Prostate	8 (19.5)	4 (19.0)	4 (20.0)
	Lung	4 (9.8)	1 (4.8)	3 (15.0)
	Other	4 (9.8)	2 (9.5)	2 (10.0)
Marital Status, n (%)				
	Married	24 (58.5)	13 (61.9)	11 (55.0)
	Divorced or separated	4 (9.8)	2 (9.5)	2 (10.0)
	Widowed	6 (14.6)	1 (4.8)	5 (25.0)
	Never married	7 (17.1)	5 (23.8)	2 (10.0)
Employment status, n (%)				
	Working full-time	15 (36.6)	10 (47.6)	5 (25.0)
	Retired	19 (46.3)	10 (47.6)	9 (45.0)
	Other	7 (17.1)	1 (4.8)	6 (30.0)
Current health, n (%)				
	Excellent	6 (14.6)	5 (23.8)	1 (5.0)
	Very good	15 (36.6)	6 (28.6)	9 (45.0)
	Good	13 (31.7)	6 (28.6)	7 (35.0)
	Fair	5 (12.2)	4 (19.0)	1 (5.0)
	Poor	1 (2.4)	0 (0)	1 (5)
	No response	1 (2.4)	0	1
Computer access, n (%)				
	Dial-up or low speed	1 (2.4)	1 (4.7)	0 (0)
	High speed	39 (95.1)	19 (90.5)	20 (100.0)
	No response	1 (2.4)	1 (4.8)	0 (0)
Computer use, n (%)				
	Regular	35 (85.4)	18 (85.7)	17 (85.0)
	Occasional	3 (7.3)	2 (9.5)	1 (5.0)
	Rare	1 (2.4)	0 (0)	1 (5.0)
	Never	1 (2.4)	0 (0)	1 (5.0)
	No response	1 (2.4)	1 (4.8)	0
Referring clinician type				
	Radiation oncology	32 (78.0)	17 (80.9)	15 (75.0)
	Medical oncology	8 (19.5)	3 (14.3)	5 (25.0)
	Surgical oncology	1 (4.9)	1 (4.8)	0

^a^Note: individual values are rounded and may not total 100%.

After 2 months, 25 (61%) of the 41 enrolled patients provided follow-up data. We made repeated attempts to contact enrolled participants, except where early contacts resulted in expressed desire for no further participation. Reasons given for not participating in follow-up included ill health and change of residence.

Of the 25 patients who participated in follow-up (13 in the provider-initiated arm, 12 in the patient-initiated arm), 11 (44%) had initiated an SCP. In the patient-initiated arm (n=20), 8 initiated a plan, with 5 of these completing the plan and 3 of these 5 reporting that they had given the plan to their primary care provider (PCP). In the provider-initiated arm (n=21), we were not able to assess the number of plans started but not completed or provided to the patient; 3 patients had received a completed SCP by the 2-month follow-up, and 2 of these patients reported having provided the SCP to their PCP.

### Clinician Perspectives on SCP Facilitators and Barriers

In the baseline qualitative interviews, clinicians noted the *value* of SCPs in terms of (1) providing a good summary of treatment and an “exit strategy”; (2) potential to assist patient communication; (3) potential to increase both patient knowledge and a sense of empowerment; (4) utility if accurate and concise; (5) potential to save clinician time if patient initiated; and (6) valuable if they provide something additional to the clinical encounter. In total, 10 of the 13 clinicians made some comment in support of the concept of SCPs; 5 of these clinicians enrolled patients and only 2 actually completed provider-initiated plans. In the follow-up interviews, cancer providers reiterated many of the same perspectives, including: (1) assisting with improving transition of care to PCP (both coordination and communication); (2) improving patient knowledge of long-term effects; (3) improving patient general well-being by addressing key concerns; (4) facilitating patient investment and empowerment; (5) providing a good template for multispecialty care teams; and (6) allowing the PCP to be the “survivorship director.” There was one clinician who questioned the value of SCPs at both baseline and follow-up. Overall, however, the follow-up interviews revealed 2 main areas of potential value for clinicians: (1) improving the transition to the PCP and (2) improving patient knowledge of potential long-term effects of their treatment.

At baseline, clinicians noted a variety of *facilitators* for successful implementation of SCPs, including that: (1) plans must be concise and easy to use and understand; (2) there should be a staff member (such as a clinical nurse) dedicated to the task of delivering the plan; and (3) the timing of the plan is important. There was considerable disagreement between oncologists about the optimal time to initiate a SCP; some felt that plans should be provided earlier, whereas others said that they would like to see plans provided later in the care trajectory to avoid overwhelming patients during their cancer care. The providers also noted that patients may not be sufficiently knowledgeable about their disease and treatment to accurately complete the SCP sooner. Facilitators most often mentioned at follow-up were patient engagement/motivation, having dedicated staff, plans being concise, and use of electronic medical records (EMRs) for easy access to patient treatment data between settings.

At baseline, the primary *barriers* identified by the clinicians were clinician time and patient knowledge. In initial interviews, clinicians most often discussed a preference for patient-initiated SCPs. At follow-up, the main points highlighted by the clinicians regarding barriers were clinician time, patient engagement or motivation, patient knowledge, not having dedicated staff, over-complicated plans, and the use of EMRs to the extent that time spent inputting information reduced time available for interaction with the patient. In addition, clinicians expressed support for the idea that there should be strong patient buy-in for survivorship care planning, such that there was some perception that plans that are patient-initiated might be more successful. Another point raised was that when plans are patient-initiated, there is potential for saving time for the clinician.

### Patient Informational and Support Needs and Preparedness for Survivorship

Results from the CaSUN needs assessment at baseline indicated few unmet needs (average > 2.0) in either intervention arm (Table 2). In both the provider- and patient-initiated arms, the areas of unmet needs were *feeling like I am managing my health together with the medical team*, *knowing that all my doctors talk to each other to coordinate my care*, and *managing concerns about the cancer coming back*. In the patient-initiated arm, additional areas of unmet need were *needing local health care services* and *an ongoing case manager*. There was only one statistically significant difference between arms at baseline: *needing local health care* (*P*=.03). At follow-up, patients in both arms continued to report unmet needs in knowing that my doctors talk to each other to coordinate my care and managing concerns about the cancer coming back. Patients in the patient-initiated arm also continued to report unmet needs in *managing my health together with the medical team*, although this was no longer the case for patients in the provider-initiated arm.

**Table 2:** Cancer Survivors’ Unmet Needs at baseline and follow-up by intervention arm.

Mean (Standard Deviation)^a^	Provider-initiated at baseline (n=21)	Patient-initiated at baseline (n=20)	*P* value for differences at baseline between arms^b^	Provider-initiated at follow-up (n=13)	Patient-initiated at follow-up (n=12)	*P* value for differences at follow-up between arms^b^
I need up to date information	1.8 (0.5)	1.5 (0.6)	.12	1.8 (0.9)	2.0 (1.3)	>.99
My family and/or partner needs information relevant to them	1.7 (0.8)	1.6 (0.7)	.70	1.3 (0.5)	1.6 (0.9)	.70
I need information provided in a way that I can understand	1.6 (0.5)	1.9 (1.0)	.66	1.6 (0.5)	2.0 (1.1)	.56
I need the very best medical care	1.8 (0.4)	2.0 (0.3)	.11	1.7 (0.5)	2.0 (0.8)	.45
I need local health care services that are available when I require them	1.5 (0.5)	2.1 (1.0)	.03	1.6 (0.5)	1.9 (1.4)	.91
I need to feel like I am managing my health together with the medical team	2.1 (0.9)	2.2 (0.8)	.61	1.7 (0.7)	2.3 (0.9)	.13
I need to know that all my doctors talk to each other to coordinate my care	2.2 (1.3)	2.6 (1.2)	.08	2.3 (1.1)	2.9 (1.5)	.34
I need any complaints regarding my care to be properly addressed	1.8 (0.9)	2.0 (1.0)	.46	1.5 (0.5)	1.6 (0.9)	>.99
I need access to complementary and/or alternative therapy services	1.7 (1.0)	1.9 (1.2)	.69	1.5 (0.7)	1.6 (0.9)	.84
I need help to reduce stress in my life	1.9 (0.9)	1.8 (1.0)	.46	2.0 (1.1)	1.4 (0.5)	.15
I need help to manage ongoing side effects and/or complications of treatment	1.4 (0.5)	1.8 (1.1)	.32	1.9 (0.9)	1.7 (0.7)	.70
I need help to adjust to changes in my quality of life as a result of my cancer	1.8 (1.0)	2.0 (1.2)	.91	1.5 (0.7)	1.5 (0.7)	.90
I need help with having a family due to fertility problems	1.1 (0.2)	1.0 (0.0)	.35	1.0 (0.0)	1.0 (0.0)	-^c^
I need assistance with getting and/or maintaining employment	1.1 (0.5)	1.1 (0.2)	.58	1.0 (0.0)	1.1 (0.3)	.39
I need help to find out about financial support and/or government benefits to which I am entitled	1.4 (1.0)	1.6 (1.2)	.63	1.0 (0.0)	1.0 (0.0)	-^c^
Due to my cancer, I need help getting life and/or travel insurance	1.1 (0.2)	1.5 (0.9)	.12	1.0 (0.0)	1.2 (0.6)	.39
Due to my cancer, I need help accessing legal services	1.2 (0.9)	1.2 (0.5)	.58	1.0 (0.0)	1.0 (0.0)	-^c^
I need more accessible hospital parking	1.4 (0.8)	1.8 (1.2)	.25	1.0 (0.0)	1.2 (0.4)	.19
I need help to manage my concerns about the cancer coming back	2.6 (1.3)	2.3 (1.4)	.34	2.3 (1.3)	2.4 (1.4)	>.99
I need emotional support to be provided for me	1.8 (0.9)	1.5 (0.8)	.27	1.7 (1.1)	1.5 (0.7)	.75
I need help to know how to support my partner and/or family	1.5 (0.8)	1.7 (1.1)	.78	1.3 (0.7)	1.2 (0.4)	.88
I need help to deal with the impact that cancer has had on my relationship with my partner	1.5 (0.9)	1.6 (1.0)	.96	1.5 (0.9)	1.1 (0.3)	.22
I need help with developing new relationships after my cancer	1.2 (0.9)	1.2 (0.7)	>.99	1.1 (0.3)	1.0 (0.0)	.34
I need to talk to others who have experienced cancer	1.8 (1.1)	1.6 (1.0)	.45	1.5 (0.7)	1.4 (0.7)	.61
I need help to handle the topic of cancer in social and/or work situations	1.8 (1.1)	1.5 (1.0)	.23	1.1 (0.3)	1.4 (0.7)	.33
I need help to adjust to changes to the way I feel about my body	1.7 (1.0)	1.4 (0.9)	.13	1.5 (0.9)	1.3 (0.7)	.54
I need help to address problems with my/our sex life	1.3 (0.8)	1.6 (1.2)	.84	1.2 (0.4)	1.5 (0.7)	.40
I need an ongoing case manager to whom I can go to find out about services whenever they are needed	1.9 (1.3)	2.2 (1.5)	.57	1.5 (1.0)	1.6 (1.2)	>.99
I need help to move on with my life	1.7 (1.1)	1.7 (1.2)	.75	1.4 (0.7)	1.4 (0.7)	.81
I need help to cope with changes to my belief that nothing bad will ever happen in my life	1.5 (0.9)	1.3 (0.7)	.54	1.9 (1.1)	1.4 (0.9)	.13
I need help to cope with others not acknowledging the impact that cancer has had on my life	1.4 (0.8)	1.4 (1.0)	.58	1.8 (1.3)	1.0 (0.0)	.06
I need help to deal with my own and/or others expectations of me as a “cancer survivor”	1.6 (1.0)	1.6 (1.1)	.96	1.9 (1.3)	1.2 (0.6)	.09
I need help to try to make decisions about my life in the context of uncertainty	1.5 (0.8)	1.5 (1.1)	.84	1.7 (1.1)	1.3 (0.7)	.49
I need help to explore my spiritual beliefs	1.5 (0.6)	1.3 (0.6)	.27	1.1 (0.3)	1.1 (0.3)	>.99
I need help to make my life count	1.3 (0.5)	1.5 (0.9)	.74	1.2 (0.4)	1.3 (0.7)	.96

^a^Mean scores with 1=no need, 2=met need, 3=weak unmet need, 4=moderate unmet need, 5=strong unmet need.

^b^*P* values for Wilcoxon rank-sum tests for differences in scores between intervention arms separately at baseline and follow-up.

^c^For this question, all patients reported the same answer at follow-up, so there is no *P* value to compute.

In terms of changes on the CaSUN from baseline to follow-up (Table 3), patients in the provider-initiated arm had statistically significant improvement on *family/partner needing informatio* n (mean change: 0.5; *P*=.04), *handling the topic of cancer in social/work situations* (mean change: 0.8; *P*=.03), and *exploring spiritual beliefs* (mean change: 0.6; *P*=.04). None of the changes within the patient-initiated arm were statistically significant. There was one statistically significant difference in change between treatment arms: needing help managing my concerns about cancer improved by 0.3 in the provider-initiated arm but worsened by 0.7 in the patient-initiated arm (*P*=.03).

**Table 3:** Cancer Survivors’ Unmet Needs change from baseline to follow-up by intervention arm.

Mean (Standard Deviation)	Change^a^ in provider-initiated arm (n=13)	*P* value for change within provider-initiated arm^b^	Change^a^ in patient-initiated arm (n=12)	*P* value for change within patient-initiated arm^b^	*P* value for differences in change between arms^c^
I need up to date information	0 (0.9)	>.99	0.4 (1.0)	.34	.37
My family and/or partner needs information relevant to them	−0.5 (0.5)	.04	−0.1 (0.5)	.77	.11
I need information provided in a way that I can understand	−0.1 (0.6)	.77	0.3 (1.5)	.58	.74
I need the very best medical care	−0.3 (0.5)	.15	−0.1 (0.6)	.77	.45
I need local health care services that are available when I require them	0.1 (0.6)	.77	−0.3 (1.1)	.48	.23
I need to feel like I am managing my health together with the medical team	−0.4 (0.7)	.13	0.3 (1.0)	.41	.11
I need to know that all my doctors talk to each other to coordinate my care	−0.1 (1.2)	.89	0.4 (1.1)	.28	.37
I need any complaints regarding my care to be properly addressed	−0.2 (0.6)	.42	−0.4 (0.8)	.20	.72
I need access to complementary and/or alternative therapy services	0.0 (0.9)	>.99	−0.5 (1.2)	.27	.45
I need help to reduce stress in my life	0.2 (1.1)	.59	−0.1 (0.8)	.85	.82
I need help to manage ongoing side effects and/or complications of treatment	0.3 (1.0)	.37	0.0 (1.3)	.82	.82
I need help to adjust to changes in my quality of life as a result of my cancer	−0.1 (0.7)	.77	−0.1 (1.3)	>.99	.82
I need help with having a family due to fertility problems	0.0 (0.0)	-^d^	0.0 (0.0)	-^d^	-^d^
I need assistance with getting and/or maintaining employment	0.0 (0.0)	-^d^	0.0 (0.5)	>.99	>.99
I need help to find out about financial support and/or government benefits to which I am entitled	0.0 (0.0)	-^d^	−0.5 (1.0)	.37	.19
Due to my cancer, I need help getting life and/or travel insurance	−0.1 (0.3)	>.99	−0.5 (1.0)	.37	.56
Due to my cancer, I need help accessing legal services	0.0 (0.0)	-^d^	−0.3 (0.7)	.37	.19
I need more accessible hospital parking	−0.3 (0.7)	.37	−0.2 (0.6)	.42	.93
I need help to manage my concerns about the cancer coming back	−0.3 (0.5)	.15	0.7 (1.2)	.09	.03
I need emotional support to be provided for me	0.0 (0.7)	>.99	0.1 (0.9)	>.99	.77
I need help to know how to support my partner and/or family	−0.3 (0.7)	.37	−0.5 (1.1)	.27	.97
I need help to deal with the impact that cancer has had on my relationship with my partner	−0.2 (0.4)	.35	−0.5 (0.8)	.17	.60
I need help with developing new relationships after my cancer	0.1 (0.3)	>.99	−0.3 (0.9)	>.99	.19
I need to talk to others who have experienced cancer	−0.3 (0.7)	.23	−0.1 (0.8)	.85	.39
I need help to handle the topic of cancer in social and/or work situations	−0.8 (0.8)	.03	−0.2 (0.6)	.42	.08
I need help to adjust to changes to the way I feel about my body	−0.2 (1.0)	.71	−0.4 (0.8)	.20	.81
I need help to address problems with my/our sex life	−0.2 (0.4)	.35	−0.2 (1.3)	.71	.57
I need an ongoing case manager to whom I can go to find out about services whenever they are needed	−0.5 (1.1)	.20	−0.6 (1.1)	.11	.79
I need help to move on with my life	0.0 (0.7)	>.99	−0.3 (0.9)	>.99	.69
I need help to cope with changes to my belief that nothing bad will ever happen in my life	0.0 (0.7)	>.99	0.3 (1.0)	.59	.72
I need help to cope with others not acknowledging the impact that cancer has had on my life	0.2 (1.2)	.85	−0.3 (0.7)	.37	.46
I need help to deal with my own and/or others expectations of me as a “cancer survivor”	0.1 (1.5)	>.99	−0.2 (1.0)	.71	.68
I need help to try to make decisions about my life in the context of uncertainty	−0.1 (0.6)	.77	−0.2 (0.8)	.59	>.99
I need help to explore my spiritual beliefs	−0.6 (0.5)	.04	−0.2 (0.8)	.59	.17
I need help to make my life count	−0.1 (0.6)	.77	−0.1 (1.2)	>.99	.75

^a^Positive mean changes indicate more unmet needs; negative mean changes indicate less unmet need.

^b^*P* values for Wilcoxon signed-rank tests for change from baseline to follow-up within intervention arm, among patients with data at follow-up only.

^c^*P* values for Wilcoxon rank-sum tests for differences in the change from baseline to follow-up between intervention arms (interaction), among patients with data at follow-up only.

^d^For this question, all patients reported the same answer at baseline and follow-up, so there is no *P* value to compute.

Table 4 presents the CaSUN positive change items descriptively at baseline and follow-up by intervention arm. In the provider-initiated arm, the most frequently endorsed positive outcome at baseline was *growing as a person* (n=12 of 21; 57%). At follow-up, 9 of 13 (69%) endorsed *growing as a person* and also *benefiting from contact with other cancer survivors/families* as positive outcomes. In the patient-initiated arm, *appreciating relationships with others* more was most frequently endorsed at baseline (n=12 of 20; 60%) and at follow-up (n=7 of 12; 58%).

On the PLANS (Table 5), patients in both arms reported high survivorship knowledge and confidence. Ten of the first 11 items from the PLANS had mean scores ≥3.0, indicating that participants were between “agree” and “strongly agree” on each of the items. The only items with a mean <3.0 were knowing what to expect over the next year (mean 2.9 in the provider-initiated group) and communication with PCP (mean 2.9 in the patient-initiated group). Similarly, patients in both study arms reported high scores, on average, on the 5 “confidence” PLANS items (1=not at all confident; 10=extremely confident). In both study arms, mean scores were lowest for health care providers communicating well (8.4 provider-initiated and 7.4 patient-initiated) and highest for going to follow-up appointments (9.7 provider-initiated and 9.9 patient-initiated). There were no statistically significant differences in mean scores by intervention arm at baseline.

We also found no statistically significant differences in mean scores by intervention arm at follow-up. Again, almost all of the 11 knowledge items had mean scores ≥3.0. Among patients with follow-up in the provider-initiated group, the 2 items with mean scores <3.0 were being clear on normal symptoms (2.9) and knowing symptoms to look for (2.8). Among patients with follow-up in the patient-initiated group, the 2 items with mean scores <3.0 were communication among cancer care providers (2.8) and communication with PCP (2.7). For the 5 confidence items, scores ranged from 7.6-9.7 in the provider-initiated arm and 7.7-9.8 in the patient-initiated arm, with the same item rated lowest (health care providers communicating well) and highest (going to follow-up appointments) in both groups, similar to baseline.

**Table 4:** Cancer Survivors’ Unmet Needs positive change items at baseline and follow-up by intervention arm.

n (%)		Provider-initiated at baseline (n=21)	Patient-initiated at baseline (n=20)	Provider-initiated at follow-up (n=13)	Patient-initiated at follow-up (n=12)
I have benefited from contact with other cancer survivors and/or their families					
	*Yes, but I have always been like this*	5 (23.8)	2 (10.0)	1 (7.7)	2 (16.7)
	*Yes, this has been a positive outcome*	11 (52.4)	10 (50.0)	9 (69.2)	6 (50.0)
	*No, and I would like help to achieve this*	2 (9.5)	3 (15.0)	1 (7.7)	1 (8.3)
	*No, and this is not important to me*	3 (14.3)	5 (25.0)	2 (15.4)	3 (25.0)
I focus more on things that are important to me					
	*Yes, but I have always been like this*	10 (47.6)	9 (45.0)	5 (38.5)	6 (50.0)
	*Yes, this has been a positive outcome*	9 (42.9)	9 (45.0)	7 (53.8)	5 (41.7)
	*No, and I would like help to achieve this*	0 (0)	1 (5.0)	0 (0)	1 (8.3)
	*No, and this is not important to me*	2 (9.5)	1 (5.0)	1 (7.7)	0 (0)
I realize how precious life is					
	*Yes, but I have always been like this*	14 (66.7)	10 (50.0)	9 (69.2)	6 (50.0)
	*Yes, this has been a positive outcome*	6 (28.6)	9 (45.0)	3 (23.1)	6 (50.0)
	*No, and I would like help to achieve this*	0 (0)	0 (0)	0 (0)	0 (0)
	*No, and this is not important to me*	1 (4.8)	1 (5.0)	1 (7.7)	0 (0)
I have made lots of positive changes in my life					
	*Yes, but I have always been like this*	10 (47.6)	3 (15.0)	5 (38.5)	2 (16.7)
	*Yes, this has been a positive outcome*	6 (28.6)	8 (40.0)	5 (38.5)	4 (33.3)
	*No, and I would like help to achieve this*	2 (9.5)	4 (20.0)	1 (7.7)	2 (16.7)
	*No, and this is not important to me*	3 (14.3)	5 (25.0)	2 (15.4)	4 (33.3)
I have grown as a person					
	*Yes, but I have always been like this*	7 (33.3)	5 (25.0)	3 (23.1)	4 (33.3)
	*Yes, this has been a positive outcome*	12 (57.1)	10 (50.0)	9 (69.2)	5 (41.7)
	*No, and I would like help to achieve this*	1 (4.8)	0 (0)	0 (0)	0 (0)
	*No, and this is not important to me*	1 (4.8)	5 (25.0)	1 (7.7)	3 (25.0)
I appreciate my relationships with others more					
	*Yes, but I have always been like this*	11 (52.4)	7 (35.0)	5 (38.5)	4 (33.3)
	*Yes, this has been a positive outcome*	9 (42.9)	12 (60.0)	8 (61.5)	7 (58.3)
	*No, and I would like help to achieve this*	1 (4.8)	0 (0)	0 (0)	0 (0)
	*No, and this is not important to me*	0 (0)	1 (5.0)	0 (0)	1 (8.3)

**Table 5:** Preparing for Life as a (New) Survivor Scale at baseline and follow-up by intervention arm.

Mean (Standard Deviation)^a^	Provider-initiated at baseline (n=21)	Patient-initiated at baseline (n=20)	*P* value for differences at baseline between arms^b^	Provider-initiated at follow-up (n=13)	Patient-initiated at follow-up (n=12)	*P* value for differences at follow-up between arms^b^
I know which health care providers to call with questions about my *cancer and its treatment*	3.6 (0.7)	3.6 (0.6)	>.99	3.5 (0.7)	3.5 (0.7)	.77
I am clear which health care providers to call if I have questions about *symptoms*	3.5 (0.6)	3.5 (0.6)	.88	3.3 (0.8)	3.5 (0.7)	.66
I am clear what symptoms are normal for me to experience	3.1 (0.7)	3.2 (0.6)	.49	2.9 (0.6)	3.2 (0.8)	.37
I know what symptoms or problems I should be looking for	3.1 (0.7)	3.1 (0.7)	.94	2.8 (0.6)	3.2 (0.6)	.11
I know how frequently I should be having appointments for follow-up care	3.4 (0.6)	3.3 (0.6)	.53	3.4 (0.5)	3.2 (0.8)	.56
I am always clear about the purpose of my visits	3.3 (0.7)	3.4 (0.6)	.79	3.4 (0.5)	3.5 (0.7)	.65
I know what tests are part of my follow-up care	3.0 (0.8)	3.1 (0.8)	.77	3.3 (0.6)	3.2 (0.8)	.73
I know other things I need to do to take the best care of myself	3.3 (0.6)	3.1 (0.7)	.54	3.2 (0.6)	3.6 (0.5)	.10
The health care providers who treat me for cancer communicate well with each other	3.2 (0.9)	3.1 (1.0)	.83	3.3 (0.5)	2.8 (1.0)	.28
The health care providers who treat me for cancer communicate well with my primary care/family provider	3.3 (0.7)	2.9 (1.0)	.31	3.0 (0.5)	2.7 (1.0)	.46
I feel prepared for what to expect over the next year	2.9 (0.8)	3.0 (0.7)	.97	3.1 (0.5)	3.0 (0.9)	.97
Mean (SD)^b^						
You will call or ask questions of your health care providers when you need to	8.8 (1.5)	8.8 (2.0)	.72	8.9 (1.5)	8.6 (2.7)	.45
You will go to all your follow-up appointments	9.7 (0.7)	9.9 (0.2)	.09	9.7 (0.6)	9.8 (0.6)	.45
You will do what you need to do to take the best care of yourself	9.2 (1.0)	8.9 (1.6)	.85	8.9 (1.0)	9.2 (1.2)	.50
Your health care providers will communicate well with each other during the next year	8.4 (1.9)	7.4 (2.5)	.18	7.6 (1.2)	7.7 (2.7)	.41
There is a well-coordinated plan for your cancer care	8.7 (1.4)	8.3 (2.5)	.79	8.0 (1.4)	8.2 (2.9)	.15

^a^1=strongly disagree to 4=strongly agree.

^b^1=not at all confident to 10=extremely confident.

^c^*P* values for Wilcoxon rank-sum tests for differences in scores between intervention arms separately at baseline and follow-up.

Changes on the PLANS tended to be small in both groups (Table 6). The greatest worsening was seen in the provider-initiated group whose confidence that their health care providers will communicate well decreased by an average of 1.2 points (*P*=.01). This change was statistically significantly different from the 0.1 point improvement in the patient-initiated arm (*P*=.04 for between-group difference). No other within-group changes were statistically significant in either the provider-initiated or patient-initiated arm, nor were there any other statistically significant differences between arms.

**Table 6:** Preparing for Life as a (New) Survivor survey change from baseline to follow-up by intervention arm.

Item	Change^a^ in provider-initiated arm (n=13)	*P* value for change within provider-initiated arm^b^	Change^a^ in patient-initiated arm (n=12)	*P* value for change within patient-initiated arm^b^	*P* value for differences in change by arm^c^
**Knowledge items**						
	I know which health care providers to call with questions about my *cancer and its treatment*	0.0 (0.6)	>.99	−0.3 (0.7)	.23	.28
	I am clear which health care providers to call if I have questions about *symptoms*	−0.2 (0.7)	.48	−0.2 (0.8)	.48	.92
	I am clear what symptoms are normal for me to experience	−0.2 (0.7)	.48	0.0 (0.5)	>.99	.51
	I know what symptoms or problems I should be looking for	−0.2 (0.7)	.34	0.1 (0.5)	.77	.28
	I know how frequently I should be having appointments for follow-up care	0.0 (0.7)	>.99	−0.1 (0.9)	.85	.95
	I am always clear about the purpose of my visits	0.0 (0.6)	>.99	−0.1 (0.5)	.77	.72
	I know what tests are part of my follow-up care	0.5 (0.9)	.10	0.0 (1.0)	>.99	.21
	I know other things I need to do to take the best care of myself	0.0 (0.8)	>.99	0.3 (0.8)	.30	.30
	The health care providers who treat me for cancer communicate well with each other	−0.1 (0.5)	.77	−0.5 (1.2)	.34	.50
	The health care providers who treat me for cancer communicate well with my primary care/family provider	−0.1 (0.4)	>.99	−0.1 (0.7)	.77	>.99
	I feel prepared for what to expect over the next year	0.0 (0.8)	>.99	−0.2 (0.8)	.59	.51
**Confidence items**						
	You will call or ask questions of your health care providers when you need to	0.2 (0.4)	.35	−0.7 (2.8)	.79	.94
	You will go to all your follow-up appointments	0.1 (0.8)	.85	−0.1 (0.3)	>.99	.68
	You will do what you need to do to take the best care of yourself	−0.2 (1.5)	.72	−0.2 (0.8)	.59	.64
	Your health care providers will communicate well with each other during the next year	−1.2 (1.2)	.01	0.1 (3.3)	.63	.04
	There is a well-coordinated plan for your cancer care	−0.6 (1.5)	.16	−0.6 (2.5)	.28	.74

^a^Positive mean changes indicate improvement; negative mean changes indicate worsening.

^b^*P* values for Wilcoxon signed-rank tests for change from baseline to follow-up within intervention arm, among patients with data at follow-up only.

^c^*P* values for Wilcoxon rank-sum tests for differences in the change from baseline to follow-up between intervention arms (interaction), among patients with data at follow-up only.

### Patient Perspectives on the Benefits of SCPs and SCP Implementation

In qualitative interviews conducted at follow-up, patients expressed ongoing needs related to information and support, with almost all of those interviewed describing some ongoing negative impact of cancer in their lives. Patients discussed the emotional impacts of cancer, including depression, fatigue, anxiety, and fear. Some expressed emotions related to concerns about recurrence, and there were some descriptions of physical impacts such as pain. Even those who initially described cancer as nonimpactful tended to describe ways in which it had affected them as the interview unfolded. Several patients expressed some belief that the SCP would improve communication that would in turn help to address these concerns: *It would be a tool to communicate issues better. My biggest fear is that I know nothing about medicine.* (#27, My Care Plan; All quotes included are illustrative of broader themes to emerge from review of the patient interviews unless it is specifically noted that an idea came from just one person.)

One element of the SCP process that was seen as particularly attractive and useful for patients was “having everything in one place,” as a “quick reference document.” In general, patients seemed to like how plans made connections to health concerns other than their cancer, although not all interviewees understood why noncancer information was included on the plan. The patients who were interviewed at follow-up expressed almost universal confidence in their ability to get the care that they needed in the coming months and years. This group of patients tended to portray themselves as proactive and involved in the management of their health and health care. In some cases, patients described already having been engaged in information gathering and maintenance, but expressed that the SCP further facilitated this process. In addition to their own capacity and the value that the SCP provided, patients explained their confidence about future care with reference to their family given the quality of their health insurance and their health care providers. The SCP was seen as helping patients to identify “who to go to” and “who is responsible for what” as they moved beyond acute treatment. *I am 100% certain that I will be able to handle it. Whether I do it according to someone's protocol is another matter I will handle it to the best of my ability. As a former journalist and researcher, I am certain that I will do my research and tap all of the sources.* (#35, My Care Plan); *I feel good about it but I feel that I have to be an active participant in getting it. I have to be an active advocate—actively involved in advocating for myself. The care plan will absolutely help with this.* (#28, SCP Builder)

For the process of plan completion, several participants articulated that either putting together the plan or even simply receiving it had served to educate them about their cancer and the care received and to provide useful information that they may not have even realized they were lacking. When we asked about the potential value of the plan for improving communication with one’s PCP, patients described having existing, functional relationships with their PCPs. Patients did not mention the Web-based format of the SCP as being problematic. Their responses varied in relation to the question about whether or not they had shared their plans with their PCP, but generally patients expressed the opinion that the information provided and the format in which it was provided would be helpful in their communication with their PCP. Even patients who had not yet shared their plans with their PCP generally expressed an intention to do so. One reason provided for why they might not share a plan was that the PCP was perceived as being too busy to have time to deal with the SCP. Some patients were unsure as to whether they (or someone else) had shared the SCP with their PCP, and others expressed some uncertainty as to whether their oncologist or their internist would now act as their PCP. Patients were not universally confident that PCPs had the necessary expertise to manage their care: one patient expressed concern that their PCP would not be familiar enough with the anemia associated with his radiation treatment to effectively treat him.

In line with much of what the clinicians discussed, the timing of plan provision emerged as an important issue with patients, with several expressing a desire to have the plan earlier in the process. Several patients also noted that the plans seemed to be more summative than forward looking (planning), and some raised questions about how the plan might be updated over time. *It would have been really helpful to have more information up front.* (#25, My Care Plan); *The only question that I would have is how it gets updated over time. Does it get updated if things change? Medications? Therapy that I am undergoing… Is it a document that stands on its own? Is it reviewed annually? I don't know anything about that. It is not a static document.* (#23, SCP Builder)

In terms of content, most patients expressed satisfaction with the information provided. Areas for additional content mentioned by 1 or 2 patients included diet, contact information for specialists and information on clinical trials. A few mentioned aspects of the information provided that they did not understand; the one specific example provided was the idea of “ongoing toxicities.” There was also a potential concern expressed about the accuracy of the content provided by the providers completing the form.

## Discussion

### Principal Findings

In this mixed-methods study, we evaluated 2 models of Web-based survivorship care planning in the real-world context of 2 academically-affiliated, community hospitals. This study provides preliminary evidence of the feasibility and perceived value of 2 SCP templates, as well as possibly informing the design and implementation of future, larger studies. The combined qualitative and quantitative data provide important insights regarding the feasibility and value of the 2 SCP templates tested here, as well as survivorship care planning in general.

In terms of feasibility, a number of challenges emerged, irrespective of the study arm. First, the somewhat limited participation of eligible oncologists in this research initiative (33%) suggests that the imminent Commission on Cancer accreditation standards [17] do not provide a sufficient incentive for many oncologists to develop systems for survivorship care planning, nor does the Web-based nature or potential patient-initiated structure overcome existing barriers. Birken et al [18] identify the resources necessary for the use of SCPs as a considerable barrier to their implementation; this study does not suggest that such resource barriers are easily overcome by the mere availability of patient- or provider-initiated templates. There was higher engagement in the study (overall and in terms of actually referring patients) by radiation oncologists compared with other clinical specialties, possibly indicating an opportunity for targeted SCP initiatives. In the initial month of the study, it became clear that oncologists and their staff were experiencing considerable difficulty in identifying eligible patients for study purposes. As a result, the research team played an active role in recruiting patients, but such approaches are not sustainable for real-world applications. Even with the additional research team support for recruitment, only 5 of the 13 participating clinicians enrolled patients in the study, suggesting possible challenges in identifying eligible patients and engaging them in the process of Web-based survivorship care planning. Finally, at follow-up, fewer than half of the patients from whom we were able to collect data had access to any SCP document (including incomplete plans from the patient-initiated arm). Participants in the patient-initiated arm were more likely to have a SCP, if one included an initiated (but not completed) SCP.

Our study reveals a need for explicit consideration of how preparing and delivering the SCP should be integrated into existing practices and routines of care provision. This finding is supported by the assertion made by Keesing, McNamara, and Rosenwax [6] that there is much work to be done to resolve the practical issues in developing and delivering Web-based SCPs that originate with the provider or the patient. If the goal is that every patient completing acute treatment for cancer should have an SCP, then it is important to acknowledge that completing an SCP can be complex and requires a considerable amount of time and resources [18,19]. Effort should be spent identifying strategies for enabling providers and their staff to be reimbursed for the time that it takes to either prepare and provide the SCP or to complete a plan that is initiated by the patient. One possible way to reduce the time burden is to look for ways that SCP templates can be integrated into EMRs and/or cancer registries such that some or much of the information is auto-populated.

These findings also inform our understanding of the value of survivorship care planning. The Web-based nature of the 2 SCP modalities did not emerge as problematic, but nor was this sufficient to overcome the feasibility challenges of providing SCPs. Although both providers and patients generally supported the importance of SCPs during the qualitative interviews, data from the CaSUN and PLANS questionnaires at baseline demonstrated that the patients who enrolled in the study had relatively few unmet needs and high *perceived* knowledge of and confidence about survivorship. However, the areas rated lowest at baseline were issues that SCPs aim to address, including communication within the cancer care team and with the patient and knowing what to expect after treatment completion. In addition, at the time of transition, survivors may not know what they do not know. For example, follow-up scores were statistically significantly worse in the provider-initiated group regarding confidence health providers will communicate well with each other. In a study of SCPs for endometrial cancer survivors in the Netherlands, Nicolaije et al [20] found that patients in the SCP arm had more concerns, reported more symptoms, and experienced a greater emotional impact than control patients. Survivorship care planning may create awareness of issues that had not previously existed and uncertainty may emerge as time passes from the end of treatment. Cheung et al [21] noted that both patients and physicians may have a reason to avoid engaging in survivorship care planning discussions because of the difficult issues about long-term impacts of cancer and possible psychosocial challenges to survivorship. It is possible that this contributes to ambivalence on the part of physicians to prioritize SCP provision, at least for some patients, although this is not directly observable from our data.

In interpreting these findings, it is important to note that the 2-month follow-up was designed to evaluate delivery of the plan, not the impact of it on the secondary patient-reported outcome measures. This study was conducted in only 2 hospitals and is intended to provide preliminary evidence of the feasibility and possible value of these 2 Web-based survivorship care planning approaches and to inform the design of larger studies. The sample size was determined based on feasibility and the analysis of the quantitative data intended to inform power calculations for future studies. However, given the small numbers, the results should be applied cautiously if used to support power calculations. Statistically significant *P* values should be interpreted with caution, given the large number of tests performed. Because randomization occurred at the patient level, with providers managing patients in both study arms, it may have been more difficult for providers to establish standard processes than if they had only had to address one approach to survivorship care planning. We did not collect any patient-specific data in our follow-up interviews with clinicians, and we therefore are not able to explore whether plans were initiated (or completed), but not delivered to some patients in the provider-initiated arm. Future studies should capture process-specific data to better determine specific places or issues where any system to get SCPs to patients meets difficulties.

### Conclusion

Strengths of this study include collection of both quantitative and qualitative data from both patients and providers in 2 academically-affiliated, community hospitals. Furthermore, we used a randomized design to compare 2 SCP templates. The findings of this study provide preliminary evidence regarding the advantages and disadvantages of the 2 Web-based templates, as well as issues with survivorship care planning in general, and can inform future research in larger populations with longer follow-up. In summary, the findings of this study suggest that the primary barriers to survivorship care planning are not the templates (the *forms*), but rather the processes for completing SCPs (the *function*).

## Appendix

Multimedia Appendix 1 Journey Forward Care Plan Builder Template.

Multimedia Appendix 2 Journey Forward My Care Plan Template.

Multimedia Appendix 3 CONSORT-EHEALTH checklist V1.6.2 [21].

## Abbreviations

CaSUN: Cancer Survivor’s Unmet Needs
EMR: electronic medical records
IOM: Institute of Medicine
PLANS: Preparing for Life as a (New) Survivor
PCP: primary care provider
SCP: Survivorship Care Plan
