# Supplementary material for: Comparing Web-Based Provider-Initiated and Patient-Initiated Survivorship Care Planning for Cancer Patients: A Randomized Controlled Trial
Source: JMIR Cancer. 2016 Aug 30;2(2):e12. doi: 10.2196/cancer.5947 (PMC5369624; doi:10.2196/cancer.5947)
Supplement: Multimedia Appendix 1 [file cancer_v2i2e12_app1.pdf]

Cancer Survivorship Care Plan **SAMPLE**

This Survivorship Care Plan will facilitate cancer care following active treatment. It may include important contact information, a treatment summary, recommendations for follow-up care testing, a directory of support services and resources, and other information. [1]

Survivorship Care Plan for Breast Cancer

Prepared by: Martha Trout on 6/17/2012 at Pleasant Valley Cancer Center

General Information

**Patient Name**	Jane Plummer
**Date of birth**	6/17/1965
**Age at diagnosis**	47
**Support contact**	Tom Plummer

**Care team**	
**Hematologist/oncologist**	Dr. Howard
**General/breast surgeon**	Dr. Jones
**Radiation oncologist**	Dr. Smith
**Plastic surgeon**	None
**Primary care physician**	Dr. Wells
**OB-GYN**	Dr. Innes
**Nurse/nurse practitioner**	Martha Trout
**Mental health/social worker**	Susan Green
**Coordination of care**	

Background Information

**Family history**	None
**Genetic testing**	Not applicable
**Other health concerns**	History of cervical cancer at age 29
**Echocardiogram or MUGA result**	EF = 82%
**Additional comments**	Had annual mammogram in April 2011 which showed cyst only. Found lump under arm in August while out fishing. Jane elected to have bilateral mastectomies due to her history of cystic lumps in both breast.

© 2013 Journey Forward. All Rights Reserved. Journey Forward thanks Martha Trout MPA, BSN, OCN (R) for her content contribution.

Left breast	
Definitive breast surgery	Mastectomy on 1/12/2011
Sentinel node biopsy	No
Axillary dissection	Yes, 1/12/2011
Lymph nodes	13 removed, 3 positive
Tumor type & stage	Infiltrating ductal, T3 (Tumor is more than 5 cm (2 inches) across), N2 (Cancer has spread to 4 to 9 lymph nodes under the arm, or cancer has enlarged the internal mammary lymph nodes)
Pathologic stage	Stage III (Locally advanced cancer with small, moderate or large tumor and lymph node involvement)
ER status	Positive
PR status	Positive
HER2 status	Positive

Treatment Plan & Summary

Patient's height	64 in	
	**Pre-treatment**	**Post-treatment**
Patient's weight	127 lb	120 lb
Patient's BSA	1.61 m^2	1.57 m^2
Patient's BMI	21.8	20.6
Date last menstrual period	9/24/1994	
Comments	Due to size of tumor in breast and painful, matted nodes under arm, neo adjuvant selected. During work-up for possible clinical trial, a CT scan was done with no evidence of metastatic disease.	Skin on left chest wall was excoriated and needed dressings with silvadene until healed.

Regimen	**Paclitaxel, Carboplatin**
Treatment on clinical trial	No

Therapeutic agents	# cycles	% dose reduction
Paclitaxel	6	0
Carboplatin	6	0

Biologic therapy	Planned: Yes, Administered: Yes

**Ejection fraction**	Pre-biologic therapy: 84% (9/2/2010),<br>Most recent: 82% (12/23/2011)
**Biologic therapy treatment dates**	9/27/2010 -12/23/2011
**Pre-operative chemo administered**	Yes
**Chemotherapy treatment period**	9/27/2010 -1/3/2011
**Possible side effects of regimen**	Anemia, Cardiac symptoms, Fatigue, Hair loss, Infertility, Low blood count, Menopause symptoms, Muscle aches, Nausea/vomiting, Neuropathy
**Reconstruction**	Planned: No, Completed: No
**Radiation therapy**	Planned: Yes,<br>Completed: 3/25/2011, dose: 60 gy
**Growth factor given**	Yes
**Serious toxicities during treatment**	Neutropenia
**Hospitalization for toxicities**	No
**Neurotoxicity impairing activities**	No
**Reason for stopping chemotherapy**	Completed therapy
**Disease status at end of treatment**	Tumor shrinkage as it was given neo-adjuvantly
**Endocrine therapy**	Anti-estrogen: tamoxifen
**Endocrine therapy timing**	5 years, starting 4/11/2011

Follow-Up Care

UPON SCREENING, THE PATIENT HAS BEEN DETERMINED TO HAVE THE FOLLOWING ISSUE(S):

Patients – Please consult your health care provider for medical advice specific to you before using any medications, supplements, or other products, and before beginning any lifestyle program.

**Chief patient concern**	Concerns about risk of recurrence

Needs/Concerns	Suggested intervention(s)
**Concerns about risk of recurrence**	Patients need to know that these feelings are normal, and they won't cause the cancer to come back.<br>• Referral for cognitive behavioral therapy or counseling<br>• Referral to support group, e.g. American Cancer Society (www.cancer.org, 800-227-2345), Cancer Support Community (www.cancersupportcommunity.org, 888-793-9355)
**Fatigue**	• Regular physical activity (e.g., walking 20 minutes daily)<br>• Evaluation for hypothyroidism, anemia, depression

Test	Frequency	Provider to contact
**Medical history/physical exam: Yrs 1-3**	Every 3 months	Dr. Howard
**Medical history/physical exam: Yrs 4,5**	Every 6 months	Dr. Howard
**Post-treatment mammography**	Not needed	Dr. Innes
**MRI**		Provider decision

**Pelvic examination**	Every year	Dr. Innes
**Breast self-examination**	Every month	

**Preventive care recommendations**	Bone health, Colon cancer screening, Exercise
**Comments on follow-up care**	Jane worked throughout her treatment, taking time off only for her surgery. Her two grandsons were her focus on getting through treatment and surviving as she didn't want to miss a day with them.

Possible Late Effects of Treatment

You may experience the following effects after cancer treatment.

- **Surgery:** Numbness, weakness, pain, loss of range of motion (ROM), or arm swelling (lymphedema)
- **Chemo/Biotherapy:** Fatigue, ovarian failure with associated menopausal symptoms, neuropathy, cognitive dysfunction, weight gain, psychological distress, and sexual dysfunction; increased risk of leukemia (after anthracycline based therapy), osteoporosis from premature ovarian failure, increased risk of cardiac dysfunction secondary to anthracycline and/or trastuzumab.
- **Radiation:** Breast pain, fibrosis, telangectasia, atrophy, poor cosmetic outcome.
- **Hormone therapies:**
    - Tamoxifen—hot flashes, increased risk of blood clots, uterine cancer, and stroke.
    - Aromatase inhibitors—increased risk of osteoporosis and fracture.

Reprinted with permission. © 2011 American Society of Clinical Oncology. All rights reserved. www.Cancer.Net.

Life After Cancer Treatment: Managing Fatigue

"If I could stop feeling so tired all the time, I think I'd be fine.
I've started getting used to everything else."—Rosa

Some cancer survivors report that they still feel tired or worn out. In fact, fatigue is one of the most common complaints during the first year of recovery.

Rest or sleep does not cure the type of fatigue that you may have. Doctors do not know its exact causes. The causes of fatigue are different for people who are receiving treatment than they are for those who have finished.

- Fatigue during treatment can be caused by cancer therapy. Other problems can also play a part in fatigue, like anemia (having too few red blood cells) or having a weak immune system. Poor nutrition, not drinking enough liquids, and depression can also be causes. Pain can make fatigue worse.
- Researchers are still learning about what may cause fatigue after treatment.

How long will fatigue last? There is no normal pattern. For some, fatigue gets better over time. Some people, especially those who have had bone marrow transplants, may still feel energy loss years later.

Some people feel very frustrated when fatigue lasts longer than they think it should and when it gets in the way of their normal routine. They may also worry that their friends, family, and coworkers will get upset with them if they continue to show signs of fatigue.

Getting Help

Talk with your doctor or nurse about what may be causing your fatigue and what can be done about it. Ask about:

- How any medicines you are taking or other medical problems you have might affect your energy level
- How you can control your pain, if pain is a problem for you

- Exercise programs that might help, such as walking
- Relaxation exercises
- Changing your diet or drinking more fluids
- Medicines or nutritional supplements that can help
- Specialists who might help you, such as physical therapists, occupational therapists, nutritionists, or mental health care providers

Coping with Fatigue

Here are some ideas:

- **Plan your day.** Be active at the time of day when you feel most alert and energetic.
- **Save your energy by changing how you do things.** For example, sit on a stool while you cook or wash dishes.
- **Take short naps or rest breaks between activities.**
- **Try to go to sleep and wake up at the same time every day.**
- **Do what you enjoy, but do less of it.** Focus on old or new interests that don't tire you out. For example, try to read something brief or listen to music.
- **Let others help you.** They might cook a meal, run errands, or do the laundry. If no one offers, ask for what you need. Friends and family might be willing to help but may not know what to do.
- **Choose how to spend your energy.** Try to let go of things that don't matter as much now.
- **Think about joining a support group.** Talking about your fatigue with others who have had the same problem may help you find new ways to cope.

End Notes

Note 1: *Important caution.*

This is a summary document whose purpose is to review the highlights of the cancer chemotherapy treatment plan for this patient. This does not replace information available in the medical record, a complete medical history provided by the patient, examination and diagnostic information, or educational materials that describe strategies for coping with cancer and adjuvant chemotherapy in detail. Both medical science and an individual’s health care needs change, and therefore this document is current only as of the date of preparation. This summary document does not prescribe or recommend any particular medical treatment or care for cancer or any other disease and does not substitute for the independent medical judgment of the treating professional.
