# Supplementary material for: Comparing Web-Based Provider-Initiated and Patient-Initiated Survivorship Care Planning for Cancer Patients: A Randomized Controlled Trial
Source: JMIR Cancer. 2016 Aug 30;2(2):e12. doi: 10.2196/cancer.5947 (PMC5369624; doi:10.2196/cancer.5947)
Supplement: Multimedia Appendix 2 [file cancer_v2i2e12_app2.pdf]

My Care Plan

This Survivorship Care Plan will help you manage your health care after treatment for cancer. Fill in the *General Information* and *Self-Assessment* to the best of your abilities. Then, work with your oncology provider to fill in the *Treatment Summary* and *Follow-up Care* sections. Be sure to visit the Journey Forward Survivorship Library (JourneyForward.org/Library) to view and print factsheets related to your cancer, symptoms and ongoing needs, and keep these with your Care Plan. When your Plan is complete, make an appointment to review it with your primary care provider. Keep your Plan handy when talking with healthcare providers over time.

Reviewed with my oncologist ☐ **Reviewed with my primary care provider** ☐

General Information

Last updated	
Your name	
Your date of birth	

YOUR CARE TEAM	NAME & CONTACT INFORMATION
Support contact	
Primary care provider	
Hematologist/oncologist	
Surgeon	
Radiation oncologist	
OB-GYN ♀	
Nurse/nurse practitioner	
Mental health/social worker	

Self-Assessment

Check any symptoms you are experiencing. **Discuss symptom management and treatments with a healthcare professional.**

- [ ] Abdominal pain
- [ ] Changes in appetite
- [ ] Chest pain
- [ ] Chronic constipation
- [ ] Chronic diarrhea
- [ ] Cough or wheezing
- [ ] Decreased exercise ability
- [ ] Dental problems
- [ ] Difficulty breathing
- [ ] Dizziness
- [ ] Dry mouth
- [ ] Easy bruising or bleeding
- [ ] Fatigue
- [ ] Fertility concerns
- [ ] Fever and sweats
- [ ] General weakness
- [ ] Hair loss
- [ ] Hearing loss
- [ ] Heartburn/indigestion
- [ ] Hot flashes/night sweats
- [ ] Irregular heartbeat/palpitations
- [ ] Jaundice (yellowing of skin or eyes)
- [ ] Joint pain or muscle aches
- [ ] Leg pain with exertion
- [ ] Memory/concentration issues
- [ ] Negative body image
- [ ] New/changed moles or freckles
- [ ] Numbness/weakness on one side
- [ ] Pain or problems with eating
- [ ] Pain with urination
- [ ] Painful eyes
- [ ] Pins and needles or numbness
- [ ] Recurrent colds/coughs/infections
- [ ] Relationship problems
- [ ] Sexual dysfunction/lack of desire
- [ ] Shortness of breath
- [ ] Skin changes, rashes, lumps or bumps
- [ ] Sleep-wake disturbances
- [ ] Slurred speech
- [ ] Swelling of arm or leg
- [ ] Swollen lymph nodes
- [ ] Urinary incontinence
- [ ] Vision problems
- [ ] Weight gain or overweight
- [ ] Weight loss or loss of appetite

♀ WOMEN ONLY

- [ ] Abnormal vaginal bleeding
- [ ] Irregular menses (periods)
- [ ] Vaginal discharge
- [ ] Vaginal dryness
- [ ] Painful intercourse
- [ ] Premature menopause

♂ MEN ONLY

- [ ] Erectile dysfunction

	NOT PRESENT										. . . . WORST IMAGINABLE
SYMPTOM	0	1	2	3	4	5	6	7	8	9	10
Pain	○	○	○	○	○	○	○	○	○	○	○
Anxiety/worry	○	○	○	○	○	○	○	○	○	○	○
Fear of recurrence	○	○	○	○	○	○	○	○	○	○	○
Depression/sadness	○	○	○	○	○	○	○	○	○	○	○

Adapted from the UCLA Survivorship Center Medical History Intake Form.

Treatment Summary

This is a summary of your diagnosis and treatment. Most of this information can be found in your pathology report, operative report, and chemotherapy and radiation treatment summaries. **Please consult with your oncology provider.**

Diagnosis date

Type of cancer

Location of cancer

Pathologic stage

TNM staging *T* *N* *M*

Histology

Surgery

Chemotherapy regimen

Clinical trial?

Therapeutic agents	Dose	Schedule/# cycles	Dose reductions/comments

Treatment goal

Response to treatment

Serious toxicities during treatment

Ongoing toxicities

Radiation therapy (type, dose, site)

Comments

Follow-up Care

Visit the Survivorship Library (JourneyForward.org/Library) to see guidelines for follow-up care. **BE SURE TO CONSULT WITH YOUR ONCOLOGY PROVIDER TO DETERMINE THE RIGHT SCHEDULE OF FOLLOW-UP TESTS AND VISITS FOR YOU.**

FOLLOW-UP TESTS & VISITS	WHEN/HOW OFTEN?	PROVIDER TO CONTACT
**Medical oncology visit**		
**Physical exam**		
**Bone density scan (DEXA)**		
**Imaging (X-ray, CT, MRI, PET scan)**		
**Mammogram**		
**Pap smear & pelvic exam ♀**		
**PSA & rectal exam ♂**		
**Colonoscopy**		

WELLNESS	COMMENTS
☐ **Diet & nutrition**	
☐ **Exercise**	
☐ **Mental health**	
☐ **Bone health**	
☐ **Immunizations**	
☐ **Cholesterol management**	
☐ **Diabetic screening/management**	
☐ **Hypertension control**	
☐ **Smoking cessation**	

OTHER COMMENTS

MCM0001881100
